# Histological Quantitation of Brain Injury Using Whole Slide Imaging: A Pilot Validation Study in Mice

**DOI:** 10.1371/journal.pone.0092133

**Published:** 2014-03-17

**Authors:** Zhenzhou Chen, Dmitriy Shin, Shanyan Chen, Kovalenko Mikhail, Orr Hadass, Brittany N. Tomlison, Dmitry Korkin, Chi-Ren Shyu, Jiankun Cui, Douglas C. Anthony, Zezong Gu

**Affiliations:** 1 Department of Pathology and Anatomical Sciences, University of Missouri School of Medicine, Columbia, Missouri, United States of America; 2 Center for Translational Neuroscience, University of Missouri School of Medicine, Columbia, Missouri, United States of America; 3 Interdisciplinary Neuroscience Program, University of Missouri, Columbia, Missouri, United States of America; 4 Informatics Institute and Department of Computer Science, University of Missouri, Columbia, Missouri, United States of America; 5 Department of Pathology and Laboratory Medicine, Alpert Medical School of Brown University, Providence, Rhode Island, United States of America; 6 Department of Neurosurgery, Zhujiang Hospital, Southern Medical University, Guangzhou, China; St Michael's Hospital, University of Toronto, Canada

## Abstract

Quantitative assessment of serial brain sections provides an objective measure of neurological events at cellular and molecular levels but is difficult to implement in experimental neuroscience laboratories because of variation from person-to-person and the time required for analysis. Whole slide imaging (WSI) technology, recently introduced for pathological diagnoses, offers an electronic environment and a variety of computational tools for performing high-throughput histological analysis and managing the associated information. In our study, we applied various algorithms to quantify histologic changes associated with brain injury and compared the results to manual assessment. WSI showed a high degree of concordance with manual quantitation by Pearson correlation and strong agreement using Bland-Altman plots in: (i) cortical necrosis in cresyl-violet-stained brain sections of mice after focal cerebral ischemia; (ii) intracerebral hemorrhage in ischemic mouse brains for automated annotation of the small regions, rather than whole hemisphere of the tissue sections; (iii) Iba1-immunoreactive cell density in the adjacent and remote brain regions of mice subject to controlled cortical impact (CCI); and (iv) neuronal degeneration by silver staining after CCI. These results show that WSI, when appropriately applied and carefully validated, is a highly efficient and unbiased tool to locate and identify neuropathological features, delineate affected regions and histologically quantify these events.

## Introduction

Quantitative, rather than qualitative, assessment has several advantages in collecting, analyzing, interpreting, and communicating results of an investigation. For the evaluation of tissue sections, quantitative histological analyses provide more objective datasets to assess the effects of a treatment or examine the roles of molecular signaling. Results for the findings may be compared more easily with numerical biochemical or morphological data, and evaluated statistically. However, the conventional approach for manual quantitative measurement is time consuming and inherently subjective, and is, therefore, difficult to use to analyze large datasets. Moreover, for clinical diagnosis, manual measurements frequently result in intra- or inter-observer variability, and impede inter-laboratory reproducibility [1], [2], [3].

Whole slide imaging (WSI) makes possible the development of methods for quantitative assessment of histologic data of entire glass slides. WSI has two components: acquisition of digital images of the histopathology or cytopathology slides, and viewing and management of such digital images [4], [5]. Since the first generation of automated high-speed WSI in 1999 [6], this technology has evolved to the point where digitization of whole slides at near optical resolution limits of light, can occur within a relatively short time [7]. Compared to static digital images, WSI has been shown to have more benefit for educational and diagnostic purposes [8].

Interest in using WSI in a variety of settings has grown steadily in the past decade. WSI has been used for pathological diagnosis, consensus reviews, telepathology, quality assurance, evaluation of tissue microarrays, education and proficiency testing [4], [5], [9], [10]. However, there are very few reports describing WSI in experimental neuroscience studies [11], and there has been no direct, comprehensive comparison of automated WSI annotation to conventional microscopic examination.

Necrosis, hemorrhage, microglial activation and neuronal degeneration are important histologic events occurring in neurological diseases including ischemic stroke and traumatic brain injury (TBI). Following the initial events of ischemic stroke and TBI, secondary events in the brain develop in hours to days, and even weeks. Biochemical, metabolic and cellular changes observed during the secondary injury phase are frequently associated with disruption of the blood-brain barrier (BBB), intracerebral hemorrhage, edema, inflammatory responses, neuronal degeneration and cell death [12], [13]. The extent of neuronal necrosis and intracerebral hemorrhage examined with cresyl violet (CV) staining, is often used as an indicator of the severity of brain damage [14], [15]. Degenerating neuronal cell bodies, as well as axon terminals and dendrites, show a high affinity for silver (argyrophilia) compared to intact neurons, and are commonly visualized with silver-stained tissue sections [16]. Microglia are resident immune effector cells in the central nervous system, as a major source for neuroinflammatory responses associated with different types of brain injury that lead to tissue disruption and cell death [17]. Activated microglia assume a different morphology, migrate to injury sites, phagocytize cellular debris, release cytokines, and notably, up-regulate expression of the calcium binding protein Iba-1 [18]. Consequently, immunohistochemistry detection of Iba-1 is commonly used to indicate microglial activation in response to pathological insults.

In this study, we applied various image analysis algorithms including pattern recognition-based Genie classifier, positive-pixel count, nuclear morphometry, and color deconvolution to quantify the following histologic events in mice: (i) cortical necrosis in focal cerebral ischemia section; (ii) intracerebral hemorrhage in focal cerebral ischemia section; (iii) Iba1-immunoreactive microglial cell density in the brain sections after controlled cortical impact (CCI); and (iv) neurodegeneration in the silver-stained CCI-brain sections. The algorithm-derived data were compared with the manually measured results in order to assess the agreement between these two methods. Direct comparisons determined potential applications and indicated precautions of using WSI for quantitative assessment of brain injury.

## Results

### Comparison of manual annotation and Genie classification of cortical necrosis

**Figure 1:**
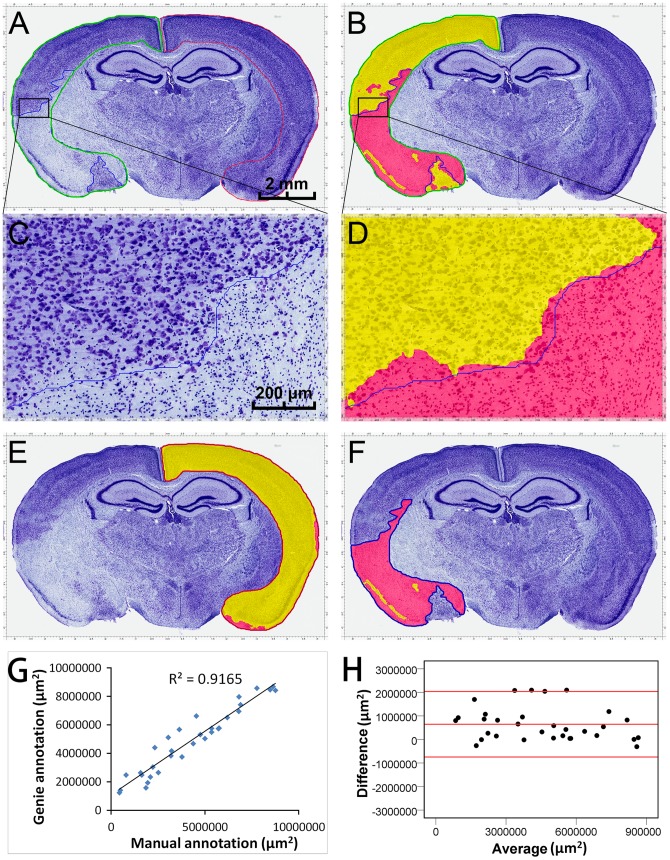
Comparison of manual annotation and Genie classification of cortical necrosis. After transient focal cerebral ischemia in mice, the cresyl-violet (CV)-stained brain sections were analyzed (**A, C**). For each tested region, the outlines in green, red and blue indicate manual annotations of the ischemic cortex, the non-ischemic contralateral cortex, and the cortical necrosis area, respectively. The Genie classification algorithm recognized necrotic (pink) and intact (yellow) areas within the ischemic cortex (**B, D**). When the contralateral intact cortices were analyzed, the Genie classification algorithm indicated 3.39%±0.61% (n = 18) FPR (**E**). As the manually annotated necrosis areas were analyzed by Genie algorithm, it revealed 95.99%±0.55% (n = 21) positive recognition rate (**F**). Pearson correlation coefficient between these two annotations (**G**; R = 0.957, P = 0.000, n = 32), and Bland-Altman difference plots (**H**) comparing the agreement of two measurements are shown. The red lines indicate mean and ±1.96 standard deviation. A, B, E, F: scale bar = 2 mm; C, D: scale bar = 200 μm.

Thirty-two CV-stained brain sections from 16 mice with embolic ischemia were employed in this analysis. CV is a basic dye that binds nucleic acids, and is particularly effective in staining the Nissl substance in the cytoplasm of neurons [19]. In this study, we used cortex as our region of interest (ROI) for analysis of necrosis. As shown in Figure 1A, embolic occlusion of the middle cerebral artery (MCA) led to considerable infarct volumes in the cortex of the affected hemisphere. At higher magnification, CV staining revealed substantial damage in neuronal morphology with irregular, condensed cell bodies with less CV-stained cytosol in the ischemic cortex (Figure 1C, lower right) compared to the round, CV-stained healthy neurons. The Genie classification algorithm automatically recognized necrotic (red) and intact (yellow) areas within the ischemic cortex (Figure 1B and 1D). To test the accuracy of this algorithm, we used it to analyze the intact cortices contralaterally to the ischemic regions, which indicated a 3.39%±0.61% false positive rate (FPR) (n = 18 sections; Figure 1E). When it was applied to the manually annotated necrosis area, the Genie indicated a 95.99%±0.55% positive recognition rate (n = 21 sections; Figure 1F).

We compared the manual and Genie annotations of areas of cortical necrosis, which showed a high degree of concordance, as confirmed by the Pearson correlation coefficient (R = 0.957, P = 0.000, n = 32; Figure 1G), although the manual annotation did not identify some small areas of “normal tissue” as recognized by the algorithm-assisted Genie annotation (Figure 1F). There was also a strong agreement between these two methods, as indicated by the lower variability in the mean difference found by the Bland-Altman difference plots (Figure 1H).

### Comparison of manual and automated annotations of intracerebral hemorrhage

**Figure 2:**
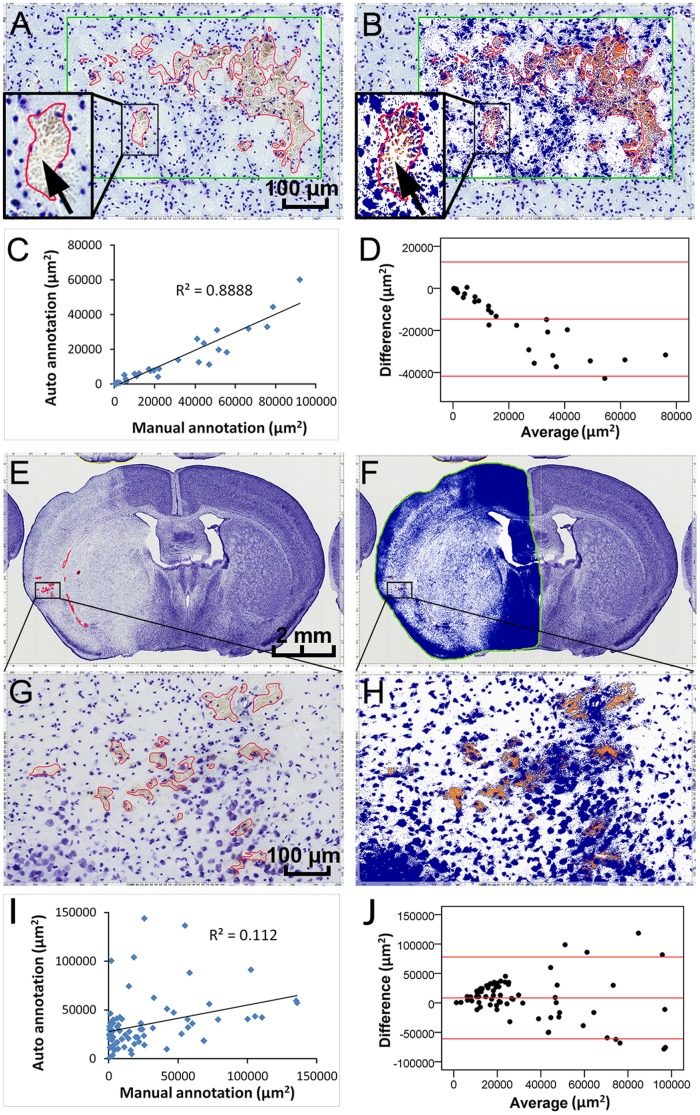
Comparison of the manual and automated annotations of intracerebral hemorrhage. The CV-stained coronal sections after focal cerebral ischemia in mice were used to examine intracerebral hemorrhage. The outlines in red and green indicate the manual annotations of the hemorrhagic areas and the regions chose for automated algorithm analysis, respectively. When the small regions were analyzed (**A**, **B**), the two annotations showed a high degree of concordance (**C**; R = 0.943, P = 0.000, n = 30), and the Bland-Altman difference plots (**D**) indicated that the automated annotations were consistently lower than the manual annotations. The arrow (**A, B,** embedded) shows that gaps between areas of blood cells, which were not excluded by manual measurement. When the whole hemisphere of the sections with hemorrhage were analyzed (**E, F; G** and **H** show an enlarged subregion of **E** and **F**, respectively), the Pearson correlation coefficient (**I**; R = 0.335, P = 0.003, n = 75) and Bland-Altman difference plots (**J**) showed low concordance and large difference between the manual and automated annotations. The red lines (in **D** and **J**) indicate mean and ±1.96 standard deviation. A, B, G, H: scale bar = 100 μm; E, F: scale bar = 2 mm.

Seventy-five sections from 17 mice with various levels of intracerebral hemorrhage in mice after embolic ischemia were employed in this analysis. CV staining revealed scattered secondary micro-hemorrhages in the ischemic area (Figure 2A). We optimized the Hue, Hue width, and color saturation threshold values before application of the positive-pixel count algorithm. The color range of manually selected micro-hemorrhage regions from three different sections were measured and converted to the 0 to 1 Hue scale, which corresponded to Hue value of 0.1 and Hue width of 0.6. Since micro-hemorrhage areas contained significant portion of the gray component, we chose a low color saturation threshold value of 0.04 to increase sensitivity of the algorithm.

We were not concerned with loss of specificity due to low color saturation threshold because the color of the counterstain was on the opposite side of the Hue circle from the Hue of the hemorrhage areas. The positive-pixel-count algorithm classified the analysis region as weak (yellow), medium (light red), strong (crimson) and non-hemorrhage (blue) (Figure 2B). We compared manual and automated analysis of minor regions with various levels of intracerebral hemorrhage (30 regions from 30 sections). The Pearson correlation coefficient showed a high degree of concordance between these two methods (R = 0.943, P = 0.000; Figure 2C). However, when we evaluated the agreement, the Bland-Altman difference plots revealed that automated annotations were frequently lower than the manual annotations. Moreover, there was an increasing tendency in the difference between these two methods with the increasing hemorrhagic area analyzed (Figure 2D).

We then applied the algorithm to those ischemic hemispheres without hemorrhage and the FPR was negligible (0.066%±0.016%, n = 12). When the whole hemisphere of the sections was analyzed (Figure 2E-2H), the Pearson correlation coefficient showed low concordance between the two methods (R = 0.335, P = 0.003, n = 75; Figure 2I). The Bland-Altman difference plots also showed weak agreement between the two methods. The differences were increased with larger hemorrhagic areas, but in contrast to the negative bias for small regions (Figure 2D), the lack of concordance was greatest at larger values, but did not show any consistent difference (Figure 2J).

### Comparison of manual and automated annotations of microglial cell density in CCI sections

**Figure 3:**
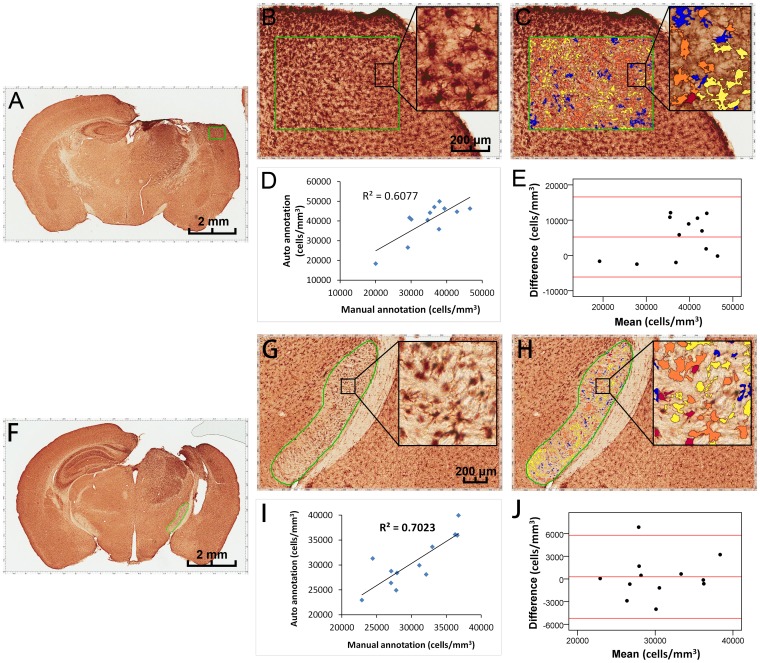
Comparison of the manual and automated annotations of microglial cell density in CCI sections. Iba-1 IHC staining was performed on the brain sections of mice subjected to CCI. A 640×480 μm^2^ subregion at the peri-lesion cortex at the section of Bregma −1.14 mm and the cerebral peduncle at the section of Bregma −2.10 mm were used as two ROIs (green outline, **A** and **F**). The Iba-1 positive microglia within the cortex have a bushy morphology with thick, densely labeled processes and large cell bodies (**B**, embedded). The algorithm produced the mark-up images (**C**), where the red, orange, and yellow pixels visualize immunoreactivity-positive cells (strong, moderate, and weak intensity, respectively), whereas blue pixels depict immunoreactivity-negative cells. Pearson correlation coefficient (**D**) and Bland-Altman difference plots (**E**) showed a high degree of concordance (R = 0.756, P = 0.004, n = 12) and strong agreement between the manual and automated annotations of microglial cell density in cortex. However, the morphology of active microglia in the cerebral peduncle was different to that in cortex, and showed an amoeboid appearance with fewer processes (**G**, embedded). With adjustment of the algorithm parameters (**H**), Pearson correlation coefficient (**I**) and Bland-Altman difference plots (**J**) showed high a degree of concordance (R = 0.838, P = 0.001, n = 12) and strong agreement between the manual and automated annotations of microglial cell density in cerebral peduncle. A, F: scale bar = 2 mm; B, C, G, H: scale bar = 200 μm.

In this study, Iba-1 immunohistochemistry (IHC) staining was performed on the sections in mice subjected to CCI, and 24 sections from 12 mice were analyzed. A 640×480 μm^2^ subregion at the peri-lesion cortex at the section of Bregma −1.14 mm and the cerebral peduncle at the section of Bregma −2.10 mm were used as two ROI (Figure 3A and 3F). Before analyses of the microglial cell density in these two ROI using manual measurement and the nuclear morphometry algorithm, we adjusted the algorithm parameters including averaging radius, curvature threshold, segmentation type intensity, and cytoplasmic rejection. For cortical regions, the size and shape of the cells from three representative areas were evaluated and a minimum nuclear size of 40 μm^2^ and minimum elongation factor 0.2 were set. The curvature threshold was kept at a low value of 2.5 to avoid coalescing nuclei. The mark-up images were reviewed to control the algorithm adjustment and were compared with the original images until optimal concordance was attained. The Iba-1 positive microglial cells at the cortex showed a bushy morphology with thick, densely labeled processes and large cell bodies (Figure 3B). The algorithm produced the mark-up images (Figure 3C), where the red, orange, and yellow pixels visualize immunoreactivity-positive cells (strong, moderate, and weak intensity, respectively), whereas blue pixels depict non-immunoreactive cells. Pearson correlation coefficient (Figure 3D) and Bland-Altman difference plots (Figure 3E) showed a high degree of concordance (R = 0.756, P = 0.004, n = 12) and strong agreement between the manual and automated annotations of microglial cell density in the cortex.

However, the morphology of active microglia in the cerebral peduncle was different than in the cortex, which had an amoeboid appearance with fewer processes (Figure 3G). We adjusted the algorithm parameters to compare the mark-up images with the original images until optimal concordance was reached. Because the cells in the cerebral peduncle regions were found to be slightly smaller than those of the cortical regions, the minimum nuclear size was decreased to 25 μm^2^ but the elongation factor was kept at 0.2 (Figure 3H). The Pearson correlation coefficient (Figure 3I) and the Bland-Altman difference plots (Figure 3J) also showed a high degree of concordance (R = 0.838, P = 0.001, n = 12) and strong agreement between the manual and automated annotations of microglial cell density in the cerebral peduncle.

### Automated assessment of neurodegeneration on silver stained CCI sections

**Figure 4:**
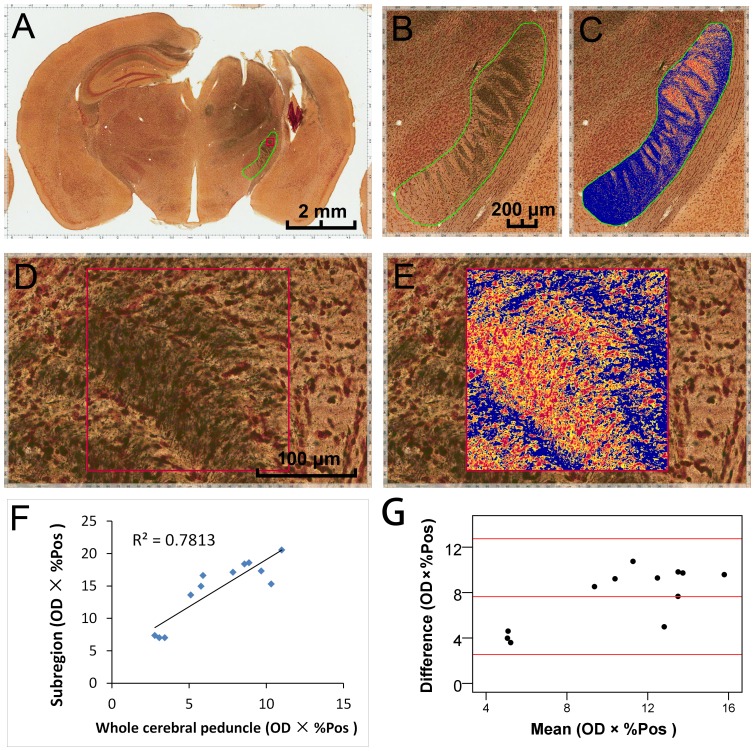
Algorithm-assisted analysis of neurodegeneration on silver stained CCI sections. Brain sections at Bregma −2.02 mm from mice subjected to CCI were processed with silver staining to reveal neuronal degeneration (**A**). The green and red outlines indicate the two ROI we chose: ipsilateral cerebral peduncle (**B**) and a 200×200 μm^2^ subregion (**D**) in the rostral region of the cerebral peduncle. Application of color deconvolution algorithm resulted in generation of mark-up images (**C**, **E**), and the optical density (OD) multiplied by the percentage of positive staining (%Pos) of these two regions were achieved, separately. Although the Pearson correlation coefficient (**F**) showed a high degree of concordance (R = 0.884, P = 0.000, n = 12) between OD × %Pos of both regions, the Bland-Altman difference plots (**G**) indicated that the data of the subregions were always higher than that of the whole cerebral peduncle. A: scale bar = 2 mm; B, C: scale bar = 200 μm; D, E: scale bar = 100 μm.

To assess neurodegeneration of TBI, twelve brain sections at Bregma −2.02 mm from 12 mice with CCI were subjected to silver staining (Figure 4A), and yielded excellent visualization of degenerating neurons and their processes [20]. To quantitatively evaluate neuronal degeneration, the color deconvolution algorithm was used to separate stains for quantification. To do so, we first measured an intensity of the gray component from three representative regions of the neuronal degeneration. The average value of the gray intensity was then converted into (0.24, 0.24, 0.24) triple in RGB color space and set as color parameters for one of the three color channels in color deconvolution algorithm settings.

We analyzed the ipsilateral cerebral peduncle as the ROI. As shown in Figure 4B, CCI led to axonal degeneration in the ipsilateral cerebral peduncle (dark-staining area, concentrated in upper right of Figure 4B). Application of the color deconvolution algorithm generated mark-up images (Figure 4C), and optical density (OD) × percentage of positive staining (%Pos) was obtained. In order to compare the WSI and the conventional method in their application on silver staining analysis, we adopted a 200×200 μm^2^ subregion in the upper part of this cerebral peduncle as another ROI (Figure 4D). The OD×%Pos values were also obtained (Figure 4E), and were compared to the data of whole cerebral peduncles. Although the Pearson correlation coefficient (Figure 4F) showed a high degree of concordance (R = 0.884, P = 0.000, n = 12) between OD×%Pos of both regions, the Bland-Altman difference plots (Figure 4G) indicated that the data of the subregions were always higher than that of the entire cerebral peduncle.

## Discussion

WSI has advantages over conventional neurohistology practice in a number of functions, such as ease of storage and retrieval, ability to share rapidly, and performance of high-throughput stereological analysis [21]. In research, it facilitates communication between the author and reader and allows for greater transparency, reproducibility and unbiased analysis [22]. Although applications of WSI technology for various disciplines are clearly successful, the validity and diagnostic accuracy of the WSI are not yet well documented in almost all pathologic fields [23]. The US Food and Drug Administration has indicated that it views WSI systems as class III (highest risk) medical devices and plans to regulate them as such [24], [25]. Therefore, validation of WSI is crucial to ensure that diagnostic performance based on digitized slides is at least equivalent to that of conventional approaches for evaluation of glass slides using light microscopy [21].

This study is the first to compare the conventional microscopic examination and WSI of quantitative assessment of brain injury in mice. We intend to validate WSI annotations in a variety of neurological events including necrosis, hemorrhage, cell identification and cell density measurement, as well as neuronal degeneration. In this study, we applied a Genie classification algorithm for detection of cortical necrosis on the CV-stained brain sections of mice after focal cerebral ischemia. This Genie-curated learning approach used manually-classified training images to generate image processing pipelines that are capable of distinguishing features of interest from the background. A previous study indicated that CV staining shows a high degree of correlation with 2,3,5-triphenyltetrazolium chloride (TTC) staining in infarct area and volume, indicating CV staining is suitable for producing accurate measurements of cerebral infarcts [19]. In the present study, a high degree of concordance and strong agreement were found between the manual and Genie annotations in the areas of cortical necrosis, suggesting the Genie recognition algorithm is suitable for detection of necrosis in cortex of the CV-stained sections. However, it should be noted that, due to the lack of CV staining in the white matter regions such as internal capsules, this Genie recognition algorithm was optimized for brain areas with a high neuronal content, such as the cortex, and because of the lack of Nissl substance or strong CV staining in white matter areas, is not suitable to detect necrosis in regions other than cortex. Other staining methods or algorithms may be adopted to overcome this limitation.

We also compared the manual and automated annotations of intracerebral hemorrhage on CV-stained brain sections of mice after focal cerebral ischemia. By optimizing the parameters of the positive-pixel-count algorithm, we were able to establish a strong correlation between the manual and automated annotations of the hemorrhage location when performed in small areas. However, the automated annotations had a negative bias in determining hemorrhagic volumes compared to manual measurement; this appeared to be due to gaps between areas of blood cells, which were not excluded by manual measurement. The difference between the two methods was even higher when larger areas of hemorrhages were analyzed. Which method is better may depend to some extent on what measurement is intended by “hemorrhage.” The automated algorithm appears to be more accurate in assessing only the bleeding volume occupied by red blood cells, while the manual method roughly defines the entire volume of brain tissue involved, including the portions of the “hemorrhage” that are not occupied by red blood cells.

When this algorithm was then run on the whole ischemic hemisphere, very low concordance and weak agreement were found between the two methods, and the differences on Bland-Altman plots were variable. When we tested the algorithm on those ischemic hemispheres without hemorrhage, the FPR was negligible. Under our experimental conditions of focal cerebral ischemia, the percentage of the total area of micro-hemorrhage to the whole hemisphere was also only 0.1354%±0.0161% in this dataset of embolic ischemia with spontaneous intracerebral hemorrhage sections. This explains why such a low FPR was associated with such large variations. Therefore, it appears that the algorithm is not appropriate when the area involved is such a small fraction of the ROI. One way to solve this shortcoming may be to reduce the ROI manually.

In this study, we also applied the IHC nuclear morphometry algorithm to calculate the microglial cell density in the CCI-induced brain sections. Transformation in cellular morphology is a characteristic change of microglia after activation. The resting microglia exhibit a typical ramified cell morphology with a small cell body, and long slender dendritic processes with secondary branching and lamellipodia [26]. Upon TBI, these thin dendritic processes retract, resulting in activated morphologies, including hypertrophic, bushy, and amoeboid patterns with thick, densely labeled processes and large cell bodies [27]. Recent work suggests that this change in morphology can be quantitatively evaluated, in various stages of microglial activation, within their native cellular context [28]. In the present study in the TBI model, Iba-1 immunoreactive microglia appeared with the usual resting morphology in sham-control animals and the contralateral hemisphere of mice with CCI (data not shown); in the ipsilateral hemisphere, they had the characteristic uniform distribution and morphologies of activated microglia. But the morphologies of these activated cells in different regions were not similar. For example in the inset of Figure 3B and 3G, the cells in the peri-lesion cortex (grey matter) showed larger cells bodies and thicker processes than those in the cerebral peduncle (white matter). Therefore, the algorithm that was suitable for the cortex should not be applied to the cerebral peduncle, as application of this algorithm setting in the cerebral peduncle tended to create large variations (data not shown). After adjusting the parameters of the algorithm, we obtained a high degree of concordance and strong agreement between the manual and automated methods in the cerebral peduncle. Taken together, it appears to be important to apply algorithms with different parameters depending on the specific cell morphologies in different regions of the brain, even on the same section.

Finally, we employed the color deconvolution algorithm to quantify the neuronal degeneration on silver-stained, CCI-induced murine brain sections. By optimizing the parameters, we were able to successfully separate the silver staining of degenerating neurons from background silver deposits. In contrast, to quantify silver staining manually, it is necessary to obtain static images of high-magnification fields and utilize image processing programs for analysis [27], [29]. Using lower magnifications, such as 1× objectives, creates static digital images of larger areas [30], but reduces the accuracy of analysis compared to higher magnifications. As a result, these tedious conventional morphometric estimates only allow measurement of OD or %Pos in selected areas, which often does not reflect the heterogeneity of argyrophilic reaction deposits. WSI circumvents this limitation by permitting the rapid analysis of entire regions of tissue sections. For example, we compared the OD×%Pos of the whole ipsilateral cerebral peduncle to a subregion in the rostral cerebral peduncle. Since the axonal degeneration mainly occurred in the rostral cerebral peduncle in these CCI-induced brain sections, the values of the subregions were always higher than that of the entire cerebral peduncle, although a high degree of concordance could be found between them.

It should be noted that the use of automated slide scanning and well-controlled procedures for histology and immunohistochemistry methods that produce consistent staining are a requirement when automating digital slide quantitation [31]. Standardization and validation of the color of digital slides on the digital display is another important aspect of digital pathology implementation. Five major reasons for color variation are thickness of specimen, staining, scanner, viewer and display [32]. Different scanners may generate differences in color appearance, even when using the same slide. Therefore, for consistent results it is necessary to optimize the algorithm parameters through a pilot sample, and validate with manual calibration before application on each batch of sections.

Because this digital pathology approach does not require expertise in image analyses, it can be adapted to a wide range of studies, and provide quantitative analysis for correlation with other biochemical data. In summary, the WSI method presented here is an important advancement for algorithm-aided analysis of neurological events, and in addition, has the ability to locate and identify particular morphological features. This tissue-based investigation process is capable of delineation and quantitation of cellular and molecular events, and provides a highly-reproducible method for pathologic assessment and creates a high-throughput capability.

## Materials and Methods

The animal experiments were approved by the Animal Care and Use Committee at the University of Missouri in accordance with the National Institutes of Health Guide for the Care and Use of Laboratory Animals. Adult male C57Bl/6J mice (The Jackson Laboratory, Bar Harbor, Maine, USA), 6–9 weeks of age and weighing 20–28 g were used in this study. Animals were housed in a 12-hour light/dark cycle and permitted food and water intake *ad libitum*. In total, 43 mice were used in this study. Mice were anesthetized with isoflurane, 5% for induction and 1.5-2% for maintenance. Rectal temperatures were maintained at 37±0.5 °C with animals placed on a thermostat-controlled heating pad (HS-3×2.5 Heater, Cell MicroControls, Norfolk, VA).

### Mouse model of embolic focal cerebral ischemia

The embolic ischemia model with the middle cerebral artery (MCA) occlusion was conducted as previously described [14]. Briefly, blood was withdrawn from the common carotid artery of a donor mouse into PE-50 polyethylene tubing, incubated at 37°C for 2 hours, and cooled at 4°C overnight to allow the formation of fibrin-rich blood clots. About 10-cm blood clots were then washed with sterile phosphate-buffered saline (PBS) in a 2-foot long PE-10 tubing for 10 rounds. After ejection from the PE-10 tubing, clots were cut into 10-mm long (0.02 μl) pieces. A single piece of clot was inserted via a PE-10 catheter with a modified tip of 0.25 mm outer diameter from the external carotid artery through the internal carotid artery into the lumen of the circle of Willis to occlude the origin of MCA. The catheter was immediately removed after injection. Relative cerebral blood flow (rCBF) was measured using laser Doppler flowmetry (MoorLab laser Doppler perfusion monitor, Moor Instruments, Devon, UK). Only animals exhibiting a >75% reduction in rCBF observed immediately after injection of a clot were included in this study for quantitative analysis.

### CCI model of traumatic brain injury in mice

CCI-induced TBI in mice by controlled cortical impact was produced with an electromagnetic impactor device as previously described [15], [33], [34]. Briefly, mice were placed on a stereotaxic frame (Benchmark Deluxe; MyNeurolab, St. Louis, MO). Following a midline skin incision, a 5.0-mm diameter craniotomy was performed in the left lateral skull using a motorized drill mounted to the stereotactic arm. The EM impactor with a 3.0 mm-diameter tip was centered at 2.7 mm lateral to midline and 3.0 mm anterior to lambda at an angle of 15° to deliver a CCI for a depth 2.5 mm below the Dura with a velocity of 0.5 m/s and dwell time of 100 ms. After injury, the impact site was covered with a plastic skull cap and the incision was sutured. Antibiotic ointment was then placed on top of the sutures and the mice were allowed to recover fully.

### Tissue processing and histological staining

At 24 hours after embolic ischemia and day 7 after CCI, time points with previously-established neurodegenerative changes [14], [15], mice were sacrificed by an overdose of isoflurane and the brains were dissected and fixed in 4% paraformaldehyde for 24 hours at 4°C. Serial 40 μm coronal sections were cut on a vibrotome (VT1200S, Leica Microsystems, Inc., Bannockburn, IL), and a total of 120–150 tissue sections were collected sequentially into a 24-well plate. For the ischemic models, every 5^th^ section was reserved for histochemical staining with cresyl violet (CV) to analyze necrosis and intracerebral hemorrhage. For the CCI models, sections at Bregma −2.02 mm were reserved for silver staining to analyze neuronal degeneration and sections at Bregma −1.14 mm and −2.10 mm were reserved for Iba-1 IHC staining to analyze the density of microglia.

Iba-1 IHC staining was performed as previously described [27]. After quenching endogenous peroxidase with 0.3% H_2_O_2_, free-floating brain sections were penetrated with 1% Triton-x-100 for 20 minutes and blocked with 2% normal goat serum in PBS containing with 0.05% Trion-x-100 for 1 hour. Sections were then incubated with 1:500 Rabbit anti-Iba-1 (Wako Chemicals USA, Richmond, VA) and 1% normal goat serum at 4°C overnight. Antibody binding was detected with biotinylated goat anti-rabbit IgG (1:500). Horseradish peroxidase method (VECTASTAIN Elite ABC PEROXIDASE KIT, PK-6101, Vector Laboratories, Burlingame, CA) and DAB were used for visualization.

For silver staining of neuronal degeneration, a FD NeuroSilver Kit II (FD NeuroTechnologies, Ellicott City, MD) was used according to the manufacturer’s instructions.

### Slide digitization and computer-aided image analysis

Using a whole slide scanner (ScanScope CS-O, Aperio Technologies, Vista, CA), the CV-stained brain sections of mice after MCA occlusion were scanned at the resolution of 0.5 μm per raw image pixel (equivalent to 20× magnification) to examine neuronal necrosis and intracerebral hemorrhage, and the CCI sections were scanned at the resolution of 0.25 μm per raw image pixel (equivalent to 40× magnification) to analyze neuronal and axonal degeneration. Images were saved in SVS format and stored in a designated local imaging Spectrum database server. The Spectrum Analysis algorithm package and ImageScope analysis software (version 11.0.2.725; Aperio Technologies, Inc.) were applied for quantitative analyses of various neurological events. The parameters of each algorithm were adjusted based on sampling images from the same batch as the main image set. This was done to avoid possible variations in staining between different batches of the slides.

Histologic Genie classifier, an automated feature detection and classification algorithm based on the statistical machine learning methods, was used for analysis of cortical necrosis as previously described [3]. GENIE is a hybrid evolutionary algorithm that addresses the general problem of finding features of interest in multispectral remotely-sensed images [35]. This type of pattern recognition-based algorithm was chosen due to its ability to detect complex morphological components of necrotic areas. Regions of cortical necrosis were manually annotated on whole slide images of the ischemic brain sections by a neuropathologist. These image areas were then used as input parameters for the Genie classifier to produce a training set. The effectiveness of the Genie training set was visualized on the randomly-selected test regions in the Aperio ImageScope viewer, which overlaid an image markup pseudo-colored for each image class. To improve accuracy, annotated image areas were adjusted by adding or removing image areas for each image class. This adjustment process was repeated until empirically optimal Genie classifier parameters were selected, and the optimized Genie classifier was then run on the ischemia images.

Regions of intracerebral hemorrhage in the CV-stained brain sections after focal cerebral ischemia can be readily distinguished by a distinct color component. Therefore, positive pixel count algorithm (Version 9.0) was chosen, using the HSI (hue, saturation, intensity) wheel that quantifies the RGB (red, blue, green) color space, for automated annotation of these areas and classified the analysis regions as weak (yellow), medium (light red), strong (crimson) and non- hemorrhage (blue). For setting thresholds of color parameters, Hue value of 0.1, Hue width value of 0.6, and color saturation threshold value of 0.04 were found to be optimal experimentally to measure pixels of hemorrhage regions on a series of test images. These values were then used for automated annotation of the main image data set.

The IHC nuclear morphometry image analysis algorithm was applied for automated annotation of Iba-1^+^ microglial cell density [11]. This type of algorithm was chosen due to its ability to capture the morphology of the stained nuclei, including cell elongation and curvature. This algorithm calculates the area of positive nuclear staining by measuring the average positive intensity (API) , optical density (OD), as well as the percentage of weak (1+), medium (2+), and strong (3+) positive nuclear staining. Iba-1^+^ microglial cell density was calculated according the formula: cell numbers in the regions of interest (ROI)/(area of ROI × 40 μm).

For evaluation of neuronal degeneration in the silver-stained tissue sections of the mouse brains with CCI, Color Deconvolution algorithm (Version 9.0) [3], [36] was used to separate an image into three channels corresponding to the actual colors of stains for quantification. The RGB OD vectors were set for each color channel. RGB OD parameter values of 0.24, 0.24, 0.24 were entered into the Color Deconvolution algorithm to define the silver stain component in the final settings. Staining was quantified by two metrics: the percentage of positive staining (%Pos), and the product of the staining OD multiplied by the percentage of positive staining (OD×%Pos). The rationale behind usage of the color deconvolution algorithm was to take advantage of its ability to separate gray color component of the degenerated regions from the color of counterstains.

### Manual annotations

Manual measurement was first completed by well-trained investigators in order to obtain a reference value prior to the automated annotations. Measurement of the necrotic area of the cortex was performed on CV-stained brain sections of mice after focal cerebral ischemia as previously described [19]. The area of micro-hemorrhage was also measured on the ischemic brain sections as previously described [14], [37]. Each serial section was carefully examined to include multiple hemorrhage areas. For manual measurement of Iba-1^+^ microglial cell density, the total positive cell number was counted and cell density (numbers of cell/mm^3^) was calculated in each ROI.

### Statistical analysis

Pearson correlation coefficient was calculated to compare the manual and automated annotations. The agreement between these two data sets was tested in Bland-Altman plots [38]. This scatterplot of the difference between methods, with reference lines at the mean difference and mean difference ±1.96×standard deviation of the differences, allows for an assessment of agreement rather than just a measure of correlation. Quantitative data were presented as mean ± standard error of mean.
